# Supplementary material for: Impact of COVID-19 on Physical Activity in Families Managing ADHD and the Cyclical Effect on Worsening Mental Health
Source: Brain Sci. 2023 May 31;13(6):887. doi: 10.3390/brainsci13060887 (PMC10296513; doi:10.3390/brainsci13060887)
Supplement: Supplementary file 1 [file brainsci-13-00887-s001.zip › brainsci-2394655-supplementary.pdf]

S1 Appendix – Parent Interview Guide

Interviewer: *Today, I am going to ask you how the COVID-19 pandemic has impacted your child and family*.

Part 1 – **Interviewer**: *When thinking of your child(ren) with ADHD…*

1) Have there been unique barriers for your child during the COVID-19 pandemic because of their ADHD diagnosis?
2) Have you noticed any changes in the presenting characteristics or symptoms associated with your child's ADHD?

Part 2 – Interviewer: *The next few questions aim to understand how the COVID-19 pandemic has impacted your child's and your physical activity participation.*

1) What was your child's daily/weekly amount of physical activity prior to the COVID-19 pandemic? (including school, extra-curricular activities)
    a. What kinds of activities were they doing?
2) What as your daily/weekly amount of physical activity prior to the COVID-19 pandemic?
    a. What kinds of activities were you doing?
3) Since the pandemic began, have you been able to incorporate physical activity into your child's/your own daily routine?
    a. If yes, how? If not, why not?
4) What is the biggest barrier to incorporating physical activity into your and your child(ren)'s routine during the COVID-19 pandemic?
5) What supports would you need to better incorporate physical activity during a pandemic?
